# Molecular Mechanism of Ethanol−Acetonitrile Azeotrope Disruption by the Ionic Liquid [BMIM][NTf_2_]

**DOI:** 10.3390/molecules31183345

**Published:** 2026-09-21

**Authors:** Pingxiao Jia, Sainan Wen, Xiuyu Du, Kuan Ji, Jiao Ma, Yilin Lyu, Yu Zhou

**Affiliations:** Shandong Sino-Japanese Center for Collaborative Research of Carbon Nanomaterials, College of Chemistry and Chemical Engineering, Qingdao University, Qingdao 266071, China; 15564849227@163.com (P.J.); 19861762065@163.com (S.W.); duxiuyu0917@163.com (X.D.); jikuanjikuan1212@163.com (K.J.); 15734950871@163.com (J.M.); 18063274669@163.com (Y.L.)

**Keywords:** ionic liquids, azeotropic separation, ethanol−acetonitrile, molecular dynamics simulation, fourier-transform infrared spectroscopy

## Abstract

Ionic liquids (ILs) have attracted significant attention as green solvents for azeotrope separation via extractive distillation; however, the microscopic mechanism by which ILs eliminate azeotropic phenomena remains inadequately understood. This study employs a combination of spectroscopic methods and theoretical calculations to elucidate the mechanism by which 1-butyl-3-methylimidazolium bis (trifluoromethanesulfonyl) imide ([BMIM][NTf_2_]) breaks the ethanol−acetonitrile azeotrope. Analysis of the hydroxyl stretching vibration region of ethanol indicates that the ethanol−acetonitrile cross-interaction is weaker than ethanol self-association, which is responsible for the minimum-boiling azeotrope. The addition of IL breaks the ethanol−acetonitrile azeotrope. The underlying mechanism is that the addition of the IL induces the formation of stronger interaction complexes between ethanol and the IL, which enhances the relative volatility of the azeotropic constituents and thereby enables azeotropic separation. This work provides a critical basis for elucidating the molecular mechanism responsible for the disruption of azeotropic phenomena by ILs.

## 1. Introduction

Ethanol is a common organic solvent widely used in medicine, chemical industry, food, energy and other fields. Acetonitrile, as a high-quality organic solvent, is widely applied in chemistry, biology, medicine, environmental science, industrial production and other fields due to its excellent chemical stability and strong solubility [1,2]. However, in industrial processes, these two organic compounds inevitably form azeotropes, which complicates their separation. Therefore, the separation of azeotropes is a significant challenge in industrial production. Currently, extractive distillation is the most commonly used method for separating azeotropes [3,4]. By adding an entrainer, the relative volatility of the azeotropic components is changed, thereby breaking the azeotropic phenomenon and achieving separation. Therefore, selecting a suitable entrainer is crucial in extractive distillation. However, traditional entrainers suffer from drawbacks such as high toxicity and low separation efficiency. Researchers have found that ionic liquids (ILs) serve as efficient entrainers for azeotrope separation [5,6,7].

ILs are salts composed of cations and anions that are liquid at room temperature. They are widely researched green solvents distinguished by their unique properties, including low melting points, stable physicochemical characteristics, and negligible volatility [8,9,10]. As entrainers in extractive distillation, ILs show great promise for separating azeotropic mixtures and have been widely applied to systems, such as alcohol−water [11,12,13], alcohol−ester [14,15,16], and acetonitrile−alcohol [17,18]. In the laboratory, vapor−liquid equilibrium experiments are usually carried out as follows: first, COSMO-RS is used to screen ILs with good separation performance, and then experiments are performed with the selected ILs [19]. It is the microscopic structures and intermolecular interactions that govern the macroscopic properties. A thorough understanding of these underlying features is essential for elucidating the mechanism by which ILs break azeotropes.

Several research groups have investigated the microscopic structures and interactions of ILs. For example, Floisand et al. employed molecular dynamics (MD) simulations to investigate the microscopic structure of dilute HOD, methanol, and ethanol in [EMIM][NTf_2_], revealing that the O−D bonds of these species interact with the [NTf_2_]^−^, while the [EMIM]^+^ interacts with the oxygen atom of the O−D group [20]. Chen et al. found that the O2 site of the [DMP]^−^ anion serves as the primary hydrogen bonding site, forming strong O−H···O hydrogen bonds with the O−H of methanol. This strong interaction is the key factor responsible for breaking the acetone−methanol azeotrope [21]. Liu et al. combined spectroscopy and theoretical calculations to demonstrate that [HMIM][OAC] breaks the ethyl propionate–ethanol azeotrope by forming stronger hydrogen bonds with ethanol than ethyl propionate’s, thus altering relative volatility and eliminating the azeotrope [22].

Although some studies have shown the critical role of specific IL−solvent interactions in azeotrope breaking, the molecular-level mechanism underlying their azeotrope-breaking ability remains poorly understood, particularly for specific systems such as ethanol−acetonitrile. Although several ILs have been reported to be effective in separating this azeotrope, the relationship between the microscopic interactions and the macroscopic separation performance has not been systematically established [23]. In particular, the role of different IL constituents (cation, anion, and their aggregates) in disrupting the ethanol−acetonitrile association remains ambiguous. Moreover, most previous studies have focused on thermodynamic phase behavior, with limited attention paid to the spectroscopic and computational identification of key interaction complexes that govern the separation. The “complex” refers to an aggregate formed by two or more molecules due to interaction energies. Yue et al. recently demonstrated that [BMIM][NTf_2_] can effectively break the ethanol−acetonitrile azeotrope at a specific concentration, but the underlying intermolecular interactions have not been elucidated [24].

In this work, we combine infrared spectroscopy, excess spectroscopy, quantum chemical calculations, and molecular dynamics simulations to systematically investigate the microscopic interaction mechanism by which [BMIM][NTf_2_] disrupts the ethanol−acetonitrile azeotrope. Specifically, we aim to identify the key interaction complexes responsible for azeotrope formation, determine how the IL competes with acetonitrile for ethanol binding, and establish a concentration-dependent mechanism for azeotrope elimination. This study provides a molecular-level basis for understanding IL-mediated azeotrope breaking and offers guidance for the rational design of IL entrainers.

## 2. Results and Discussion

### 2.1. FTIR and Excess Spectral Analysis

#### 2.1.1. IR Spectra in the *ν*(O−D) Region of the Binary System

**Figure 1:**
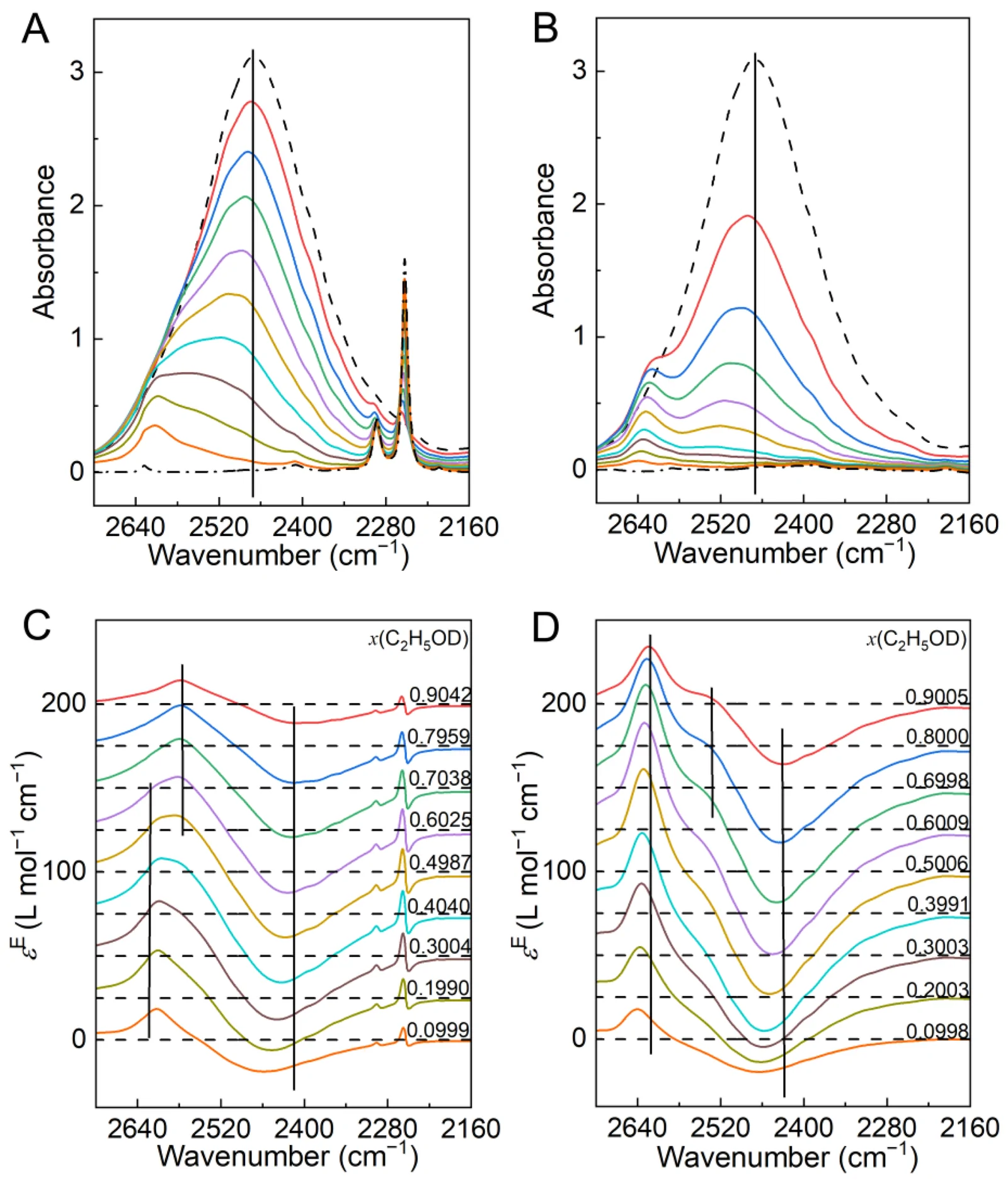
Infrared spectra (**A**,**B**) and excess infrared spectra (**C**,**D**) of the ethanol-*d*_1_–acetonitrile (**A**,**C**) and ethanol-*d*_1_–[BMIM][NTf_2_] (**B**,**D**) binary systems in the *ν*(O−D) stretching vibration region. In the IR spectra, the dashed line denotes pure ethanol-*d*_1_, while the dash dotted lines denote the pure acetonitrile (**A**) and [BMIM][NTf_2_] (**B**), respectively.

To elucidate the microstructural evolution within the ternary system, a systematic investigation of their binary systems (ethanol-*d*_1_−acetonitrile and ethanol-*d*_1_−[BMIM][NTf_2_] systems) was initiated. Figure 1A,B display the infrared spectra for the two binary systems. Analysis of the O−D stretching region (2550−2200 cm^−1^) shows a gradual reduction in peak intensity, accompanied by a clear blue shift toward higher wavenumbers, as acetonitrile or the IL is incrementally added. These spectral features indicate that the hydrogen bonding interactions formed between ethanol and acetonitrile or between ethanol and IL are inherently weaker than the self-aggregation hydrogen bonding interactions of ethanol.

**Figure 2:**
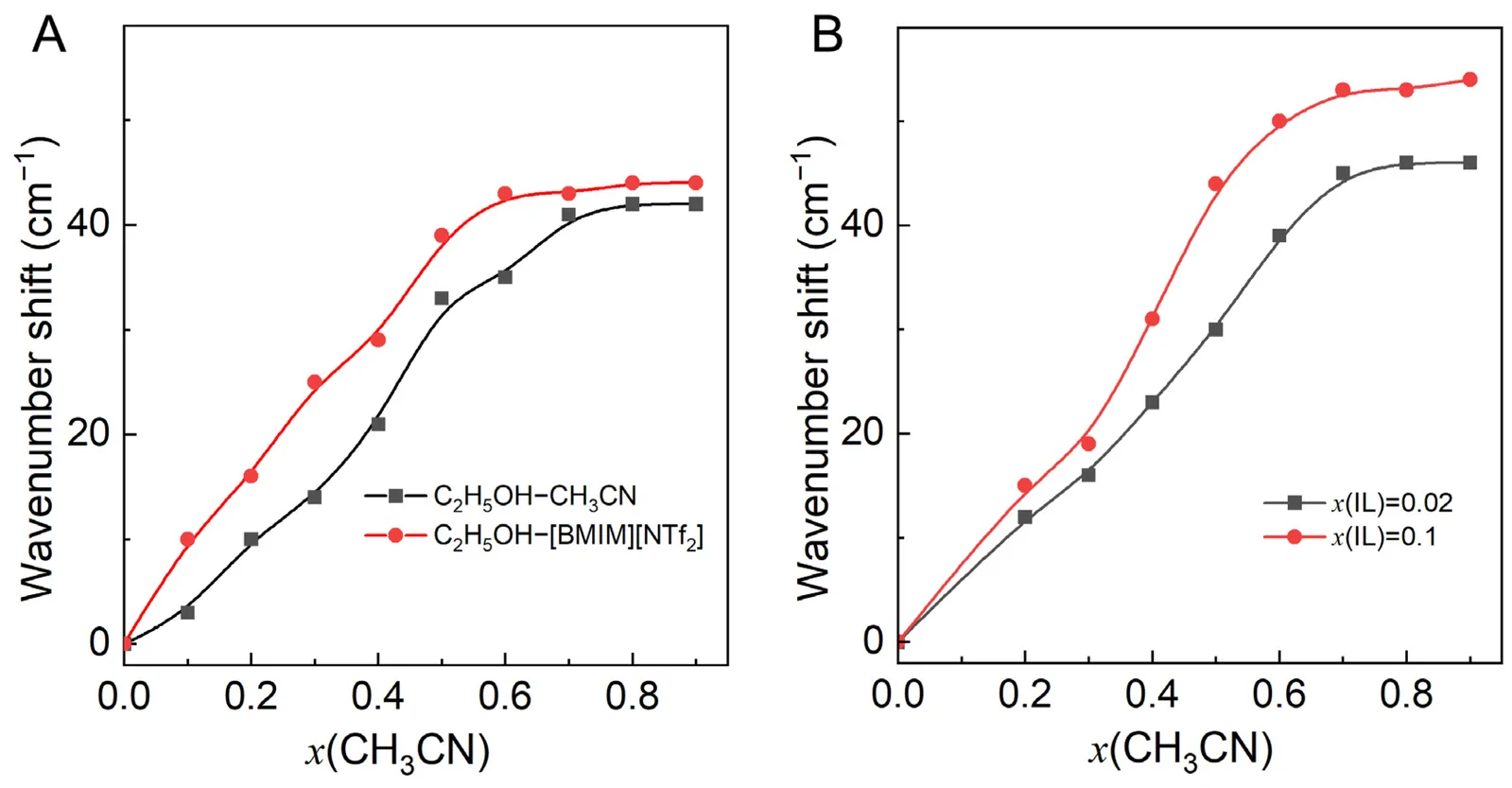
Shifts of *ν*(O−D) in the binary (**A**) and ternary (**B**) systems.

Figure 2A quantifies this blue shift [25,26], demonstrating a significantly larger magnitude upon IL addition compared to acetonitrile addition. This enhanced spectral response implies that [BMIM][NTf_2_] exhibits a stronger propensity to interact with ethanol, effectively disrupting the ethanol self-aggregation network more efficiently than acetonitrile. As shown in Figure 1A, a new shoulder band emerges at 2625 cm^−1^ when *x*(ethanol-*d*_1_) = 0.6, and its intensity progressively increases with further addition of acetonitrile.

Figure 1C,D present the excess spectra of two binary systems, respectively. Distinct positive and negative bands are observed in these spectra, indicating deviations from ideal behavior. A positive band appears at approximately 2577 cm^−1^, which is tentatively assigned to the formation of cyclic ethanol trimers–acetonitrile. Multiple negative bands are evident in the 2500−2300 cm^−1^ region, preliminarily attributed to a reduction in cyclic ethanol tetramers and larger ethanol multimers. In Figure 1C, the positive band located at 2625 cm^−1^ may be ascribed to an interaction complex formed between ethanol-*d*_1_ and acetonitrile. In Figure 1D, the positive band at 2621 cm^−1^ is correspondingly attributed to an interaction complex involving ethanol-*d*_1_ and the IL.

#### 2.1.2. IR Spectra in the *ν*(O−D) Region of Ethanol-*d*_1_−Acetonitrile−[BMIM][NTf_2_] Trinary System

**Figure 3:**
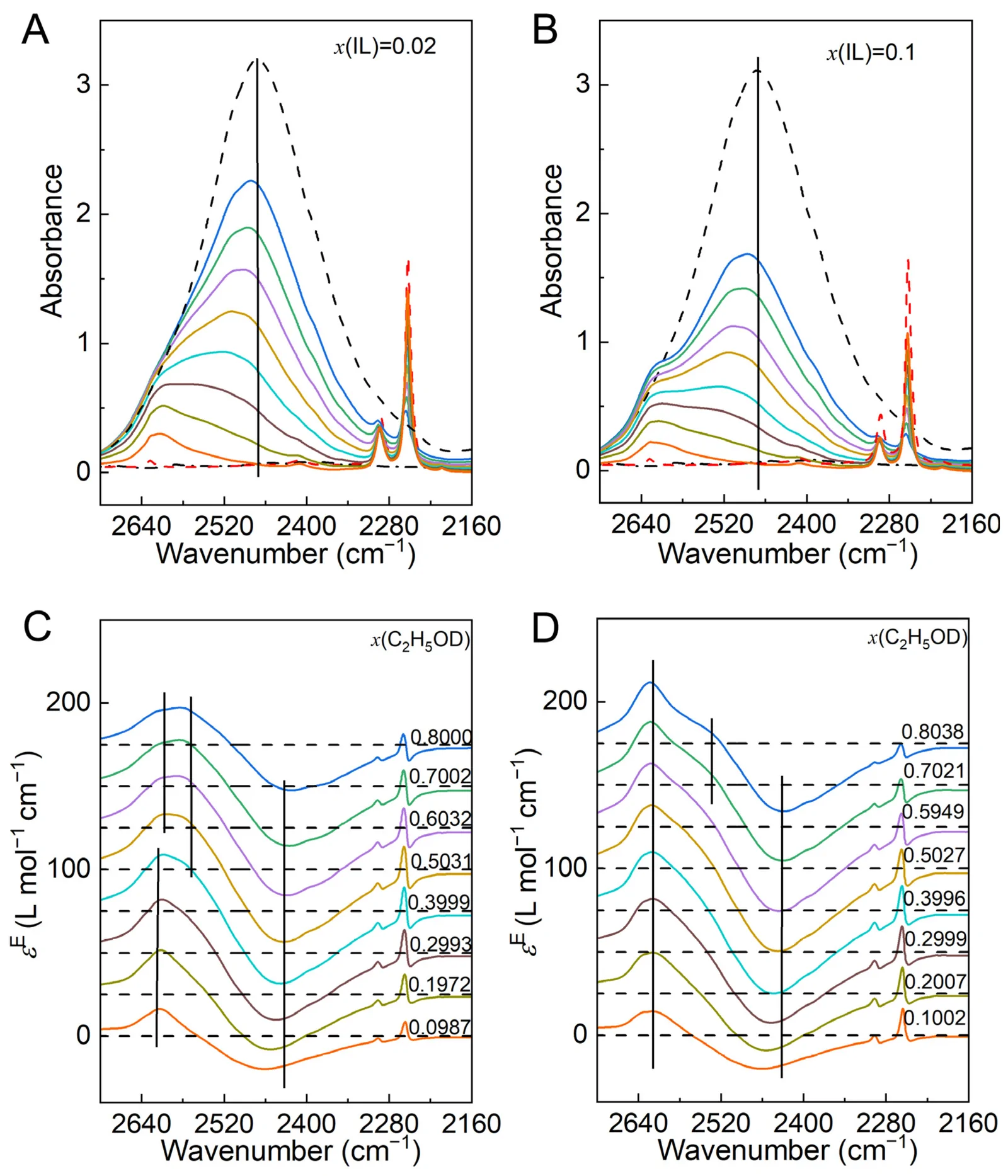
Infrared spectra (**A**,**B**) and excess infrared spectra (**C**,**D**) in the *ν*(O−D) stretching region for the ethanol-*d*_1_–acetonitrile–[BMIM][NTf_2_] system at *x*(IL) = 0.02 (**A**,**C**) and 0.1 (**B**,**D**). In the infrared spectra, the black and orange dashed lines represent pure ethanol-*d*_1_ and acetonitrile, respectively, and the black dash dotted line represents pure [BMIM][NTf_2_].

Figure 3 displays the IR spectra and excess spectra of the ternary system at fixed IL mole fractions of 0.02 and 0.1. Figure 3A,B present the IR spectra, which closely resemble those of the binary system. As acetonitrile is added, the intensity of the *ν*(O−D) band gradually decreases, accompanied by a concomitant blue shift. Figure 2B illustrates the magnitude of the blue shift of the O−D stretching vibration in the ternary system. Notably, the shift is more pronounced at *x*(IL) = 0.1, indicating that the hydrogen bonding interactions formed in the system are weaker than the ethanol self-association. A comparison with Figure 2A reveals that the blue shift is greater in the ternary system, and it increases further with higher IL concentration. This trend suggests IL exhibits a stronger disruptive effect on ethanol self-aggregates.

Figure 3C,D show the excess spectra of the ternary system. By comparison with the binary system, the band at 2620 cm^−1^ corresponds to the interaction complex between ethanol-*d*_1_ and acetonitrile, while the positive band at 2619 cm^−1^ is assigned to the complex formed between ethanol-*d*_1_ and the IL. Comparison of Figure 3C,D reveals that the interaction band corresponding to ethanol-*d*_1_–acetonitrile persists at *x*(IL) = 0.02, yet it cannot be resolved at *x*(IL) = 0.1, suggesting that this interaction complex may no longer be stable in the system, which appears consistent with the disappearance of the azeotropic behavior. Thus, we infer that the formation of the ethanol-*d*_1_–acetonitrile interaction complex appears to be an important microscopic interaction associated with azeotrope formation.

### 2.2. Theoretical Calculations

#### 2.2.1. Quantum Chemical Calculations Analysis

IR spectra revealed the presence of interaction complexes between ethanol and the other components in the system, yet their detailed nature could not be clearly described. Quantum chemical calculations were, therefore, performed to further investigate the molecular structures and interactions of these complexes. In this study, density functional theory (DFT) calculations were employed to investigate potential interaction complexes at the molecular level. A hydrogen bond is identified when the interatomic distance is less than the sum of the van der Waals radii (H⋯O < 2.5 Å, H⋯N < 2.6 Å) [27,28].

**Figure 4:**
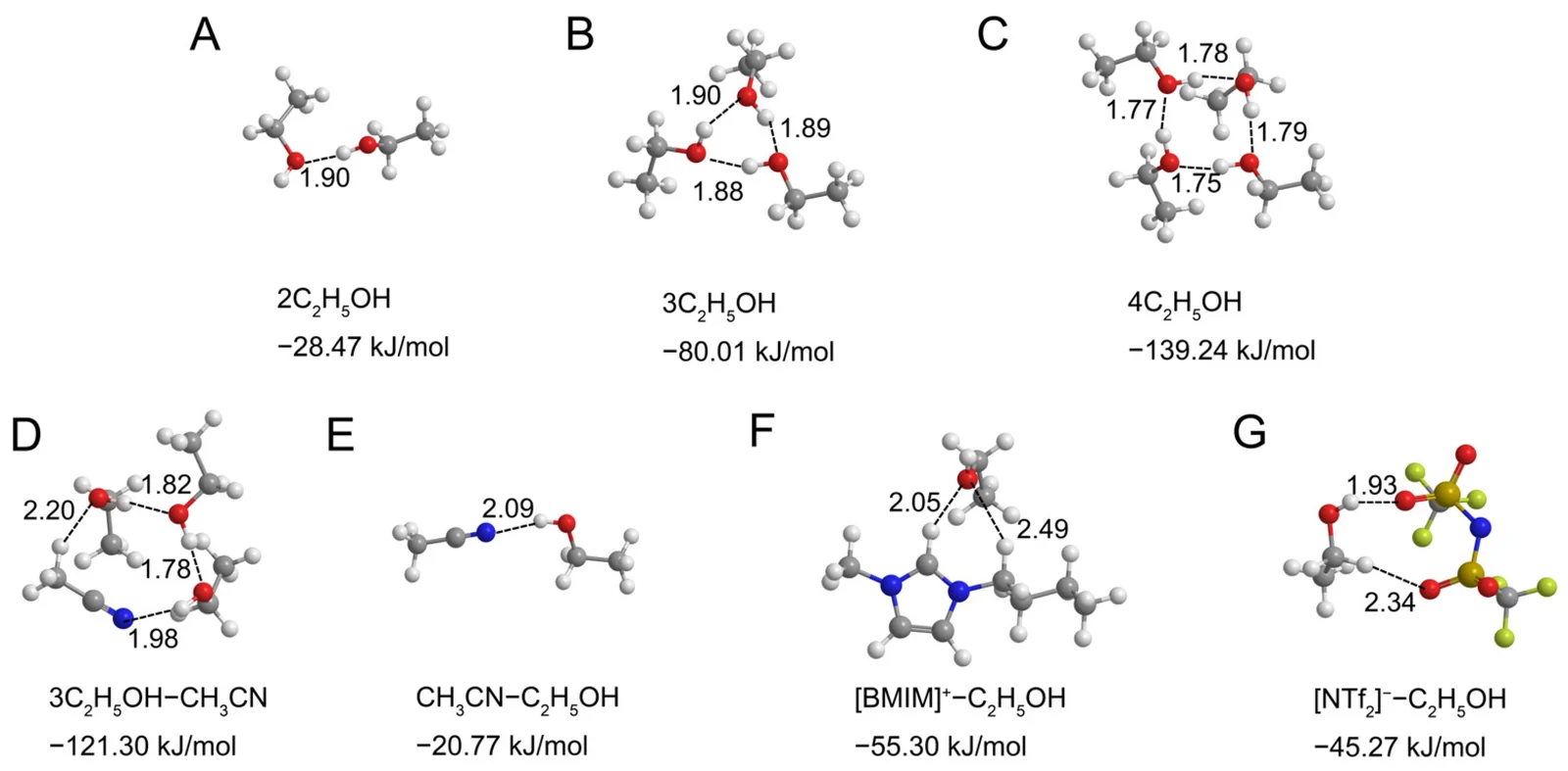
Optimized geometries of 2C_2_H_5_OH (**A**), 3C_2_H_5_OH (**B**), 4C_2_H_5_OH (**C**), 3C_2_H_5_OH−CH_3_CN (**D**), CH_3_CN−C_2_H_5_OH (**E**), [BMIM]^+^−C_2_H_5_OH (**F**), [NTf_2_]^−^−C_2_H_5_OH (**G**) complexes. The chemical elements represented by the differently colored atoms are shown in Figure S1.

**Figure 5:**
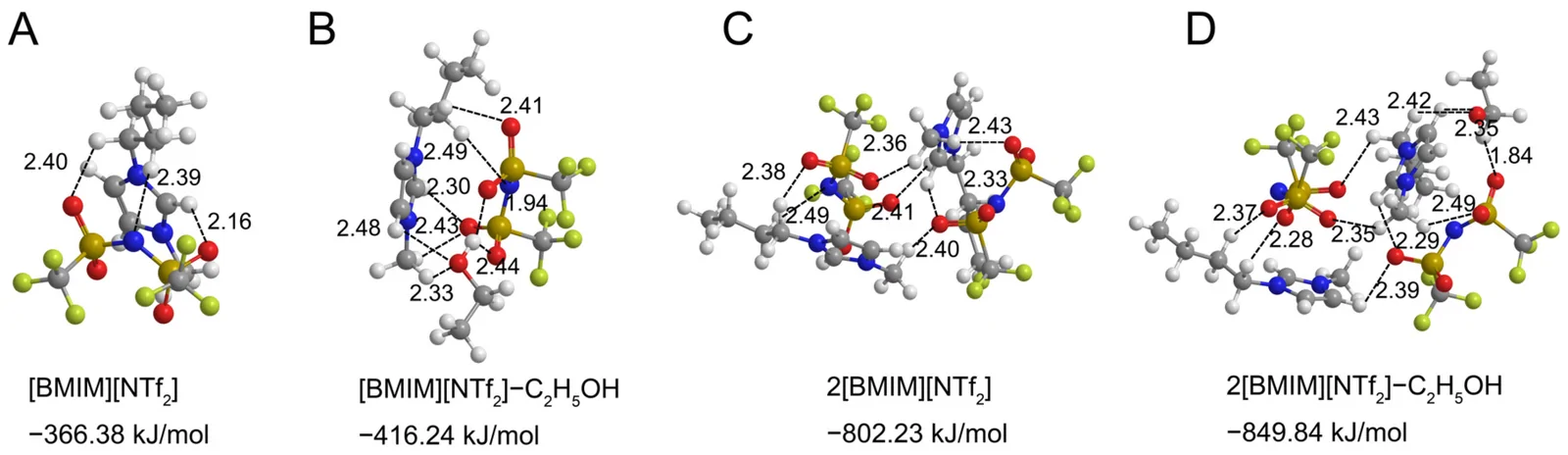
Optimized geometries of [BMIM][NTf_2_] (**A**), [BMIM][NTf_2_]−C_2_H_5_OH (**B**), 2[BMIM][NTf_2_] (**C**), and 2[BMIM][NTf_2_]−C_2_H_5_OH (**D**) complexes.

To investigate the interaction strengths of different complexes, a total of 89 configurations were optimized. Figure 4 and Figure 5 depict the most stable geometries of the possible interaction complexes, including ethanol self-aggregations, 3C_2_H_5_OH−CH_3_CN, CH_3_CN−C_2_H_5_OH, [BMIM]^+^−C_2_H_5_OH, [NTf_2_]^−^−C_2_H_5_OH, [BMIM][NTf_2_], [BMIM][NTf_2_]−C_2_H_5_OH, 2[BMIM][NTf_2_], and 2[BMIM][NTf_2_]−C_2_H_5_OH complexes.

At low concentrations, the IL exists predominantly in the form of ions. Figure 4 presents the possible interaction complexes in the solution under low conditions, including ethanol self-aggregates (Figure 4A−C) and complexes formed between ethanol and acetonitrile, [BMIM]^+^, or [NTf_2_]^−^ (Figure 4D−G). For the ethanol self-aggregates, as the cluster size increases, the hydrogen bond distance decreases and the average energy increases. Among the complexes, the interaction energy of ethanol−acetonitrile is −20.77 kJ/mol, which is significantly weaker than those of ethanol−[BMIM]^+^ and ethanol−[NTf_2_]^−^. According to the order of interaction strength, the complexes can be ranked as follows: ethanol−[BMIM]^+^ > ethanol−[NTf_2_]^−^ > ethanol−acetonitrile. This indicates that ethanol preferentially associates with the IL rather than with acetonitrile.

As the IL concentration increases, the IL may exist in the form of ion pairs and ion clusters. Figure 5A−D display the optimized structures of ion pairs, ion clusters, and their respective complexes with ethanol. According to the literature, the imidazolium-based cation primarily interacts with the anion via C2−H to form an ion pair, and two such ion pairs can further assemble into an ion cluster. As shown in Figure 5B,D, the O···H hydrogen bond distances for the ethanol−ion pair and ethanol−ion cluster complexes are 1.94 Å and 1.84 Å, respectively, indicating that ethanol also interacts strongly with ion pairs and ion clusters. These results consistently indicate that the IL−ethanol interaction is stronger than the acetonitrile−ethanol interaction. This enhanced interaction is the principal reason for azeotrope elimination in this system.

#### 2.2.2. MD Simulation Analysis

**Figure 6:**
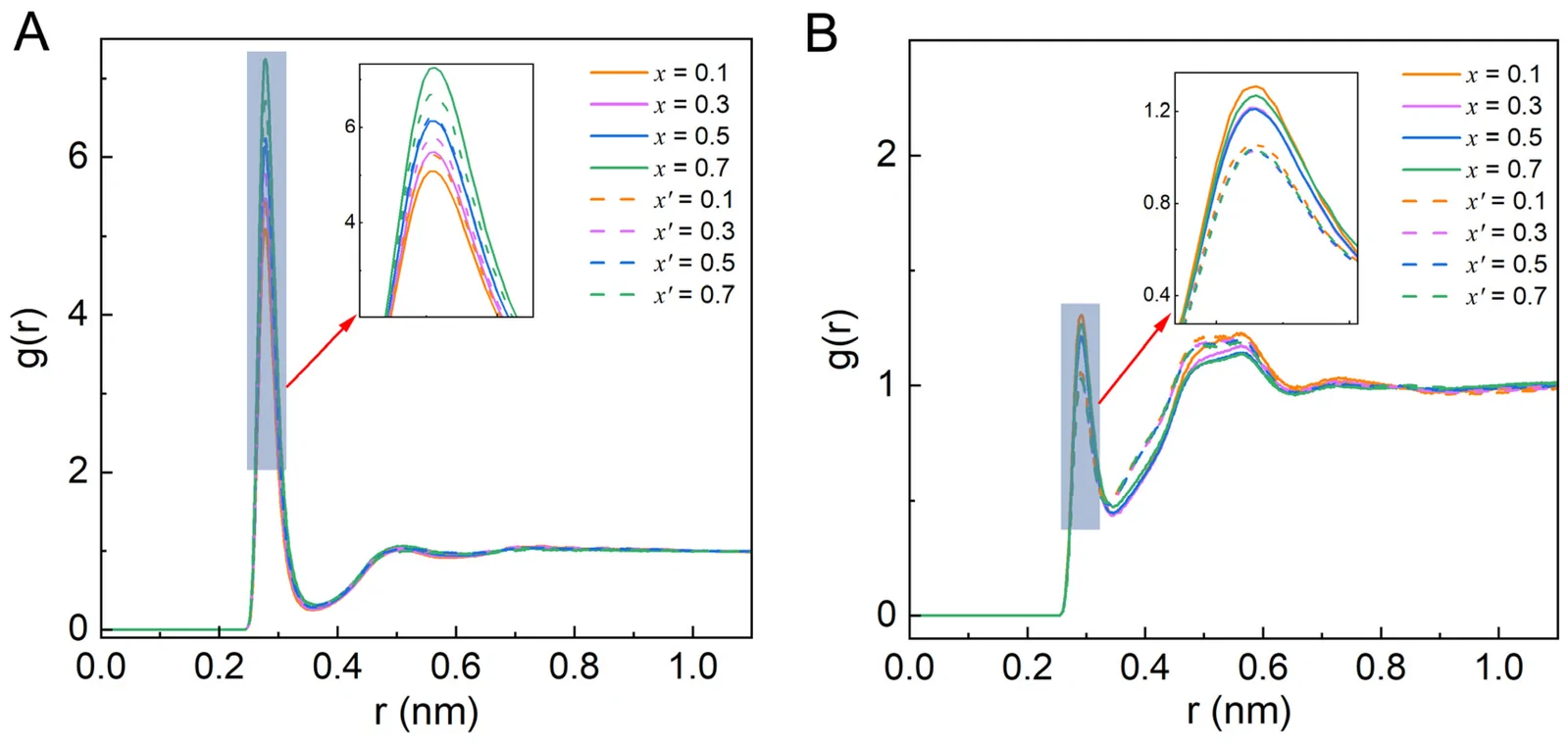
RDF of the ethanol−ethanol (**A**) and ethanol−acetonitrile (**B**) system at acetonitrile mole fractions of 0.1, 0.3, 0.5, and 0.7. The *x* denotes the absence of IL, and *x*’ denotes the RDF obtained at *x*(IL) = 0.1.

To further explore the microscopic structural properties of the binary and ternary systems, RDF and SDF calculations were carried out. In this study, the oxygen atom of ethanol was selected as the reference center, and RDFs were calculated with respect to ethanol, acetonitrile, [BMIM]^+^, and [NTf_2_]^−^. Figure 6 displays the RDFs of the ethanol−ethanol (Figure 6A) and ethanol−acetonitrile (Figure 6B), both in the absence and in the presence of the IL. In the absence of the IL, the first coordination peak of ethanol−ethanol is higher than that of ethanol−acetonitrile, indicating stronger intermolecular interactions among ethanol molecules. When the IL is present (Figure 6B), the RDF values of the ethanol−acetonitrile drop to approximately 1, which can be attributed to the relatively weak interaction between acetonitrile and ethanol.

**Figure 7:**
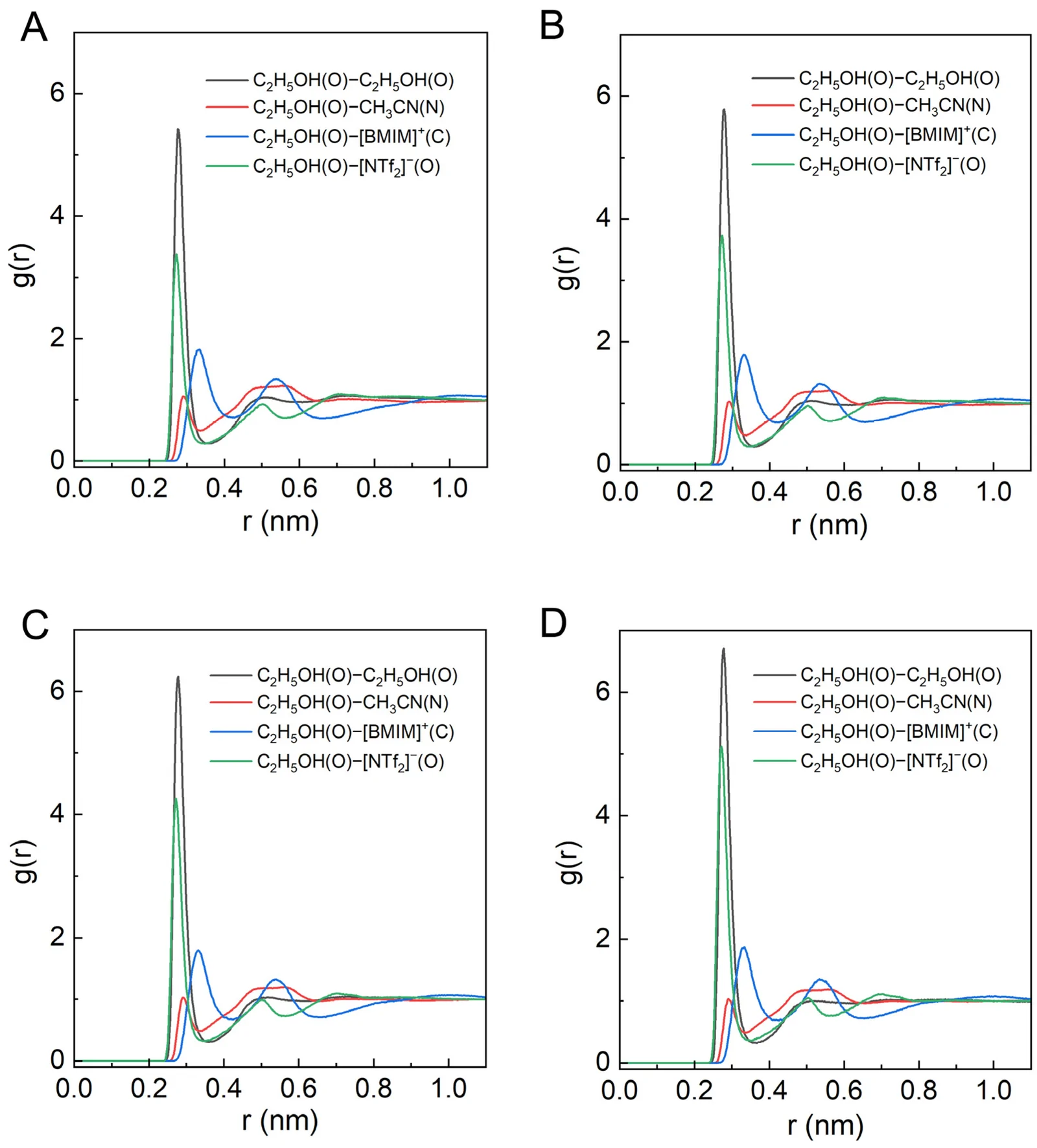
RDF between ethanol and the various components at acetonitrile mole fractions of 0.1 (**A**), 0.3 (**B**), 0.5 (**C**), and 0.7 (**D**).

The RDF can also indicate the relative intensity of interactions between two components within a system. Figure 7 presents the RDF between ethanol and the various components at different concentrations, with the IL mole fraction fixed at *x*(IL) = 0.1. With the addition of acetonitrile, the first coordination peak of the ethanol−ethanol and ethanol−anion increases continuously. At *x*(acetonitrile) = 0.1, the peak height of the ethanol−anion is 3.3-times that of the ethanol−acetonitrile; when the acetonitrile concentration reaches 0.7, the anion−ethanol peak height rises to 4.9-times that of the ethanol−acetonitrile. This further demonstrates that the ethanol−IL interaction is significantly stronger than the ethanol−acetonitrile interaction. This finding is in agreement with the results obtained from quantum chemical calculation results.

Figure S2 and Table S1 show the RDF and coordination number of ethanol−acetonitrile without and with IL at *x*(acetonitrile) = 0.1, 0.7. By analyzing the changes in coordination numbers before and after the addition of IL, it was found that the number of acetonitrile molecules around ethanol decreased significantly after the addition of IL. This decrease arises from the combined effects of dilution of acetonitrile by the IL and interactions between the IL and ethanol. Figure S3 and Table S2 show coordination numbers of ethanol, acetonitrile, [BMIM]^+^, [NTf2]^−^ around the ethanol. From the data, it can also be seen that the interaction between ethanol and the IL is stronger than that between ethanol and acetonitrile, in agreement with the RDF results.

**Figure 8:**
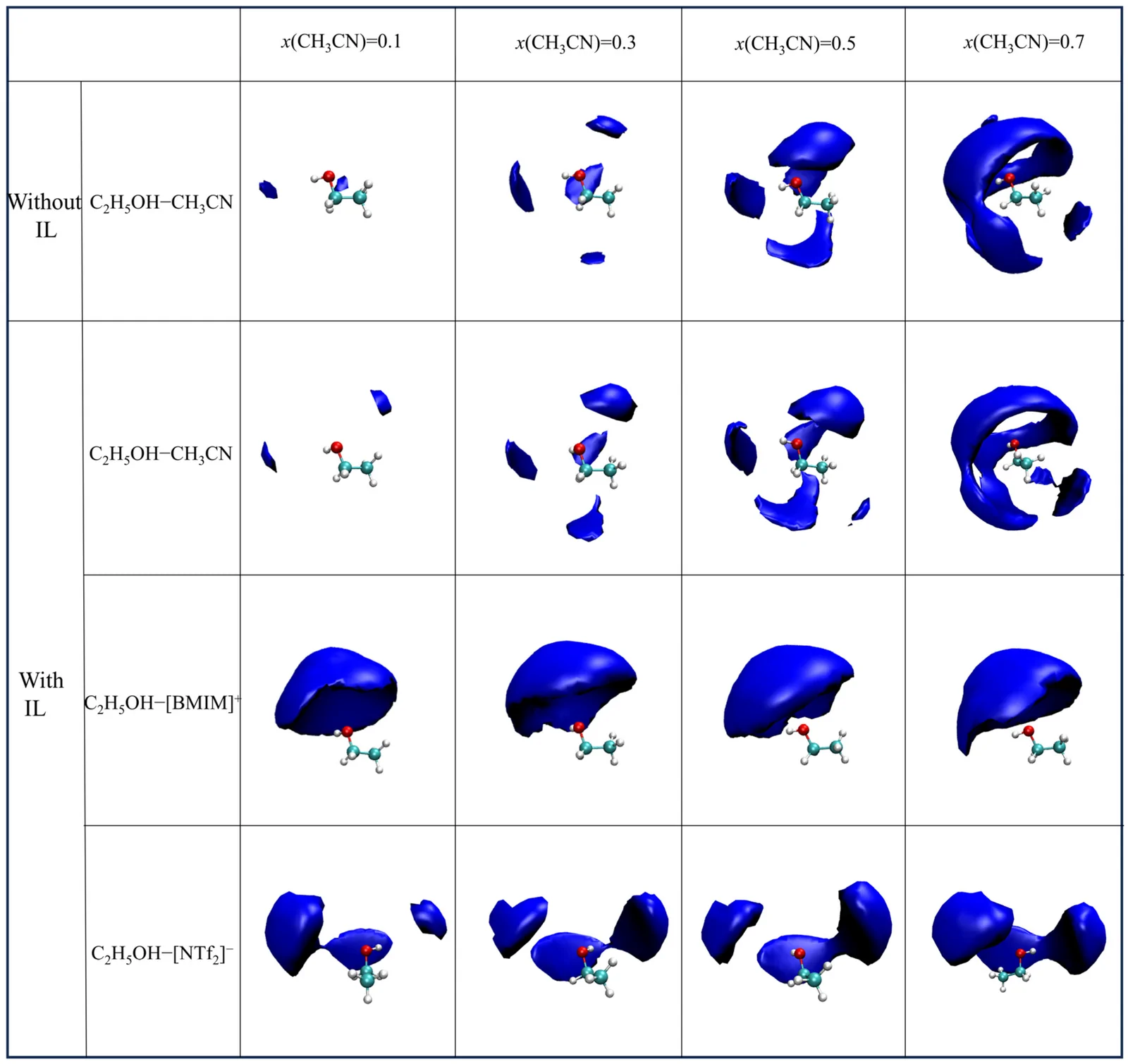
SDF of acetonitrile, [BMIM]^+^ and [NTf_2_]^−^ around ethanol in systems with the absence and presence of IL at *x*(acetonitrile) = 0.1, 0.3, 0.5 and 0.7. Different colors denote different atoms: red, O; gray, H; cyan, C.

The SDF characterizes the three-dimensional probability density distribution of a specific particle species; centered on a reference molecule, it can intuitively visualize the spatial distribution of other atoms around a given molecule in terms of isosurfaces. Figure 8 illustrates the distribution of other molecules around ethanol at different concentrations both without and with IL. In the absence of IL, the local density of acetonitrile around the ethanol’s O−H group increases with the bulk concentration of acetonitrile. In the system with IL, the acetonitrile remains present around ethanol. And the IL tends to accumulate preferentially in closer proximity to the O−H group. This behavior likely arises from stronger interactions of the cation and anion at the hydroxyl site. Moreover, with increasing acetonitrile concentration, the spatial distributions of [BMIM]^+^ and [NTf_2_]^−^ near the O−H group become broad and relatively uniform, exhibiting minimal variation under different conditions. This spatial preference of the IL components indicates the formation of stronger interaction complexes between ethanol and the IL.

### 2.3. Assignment of ν(O−H)

**Figure 9:**
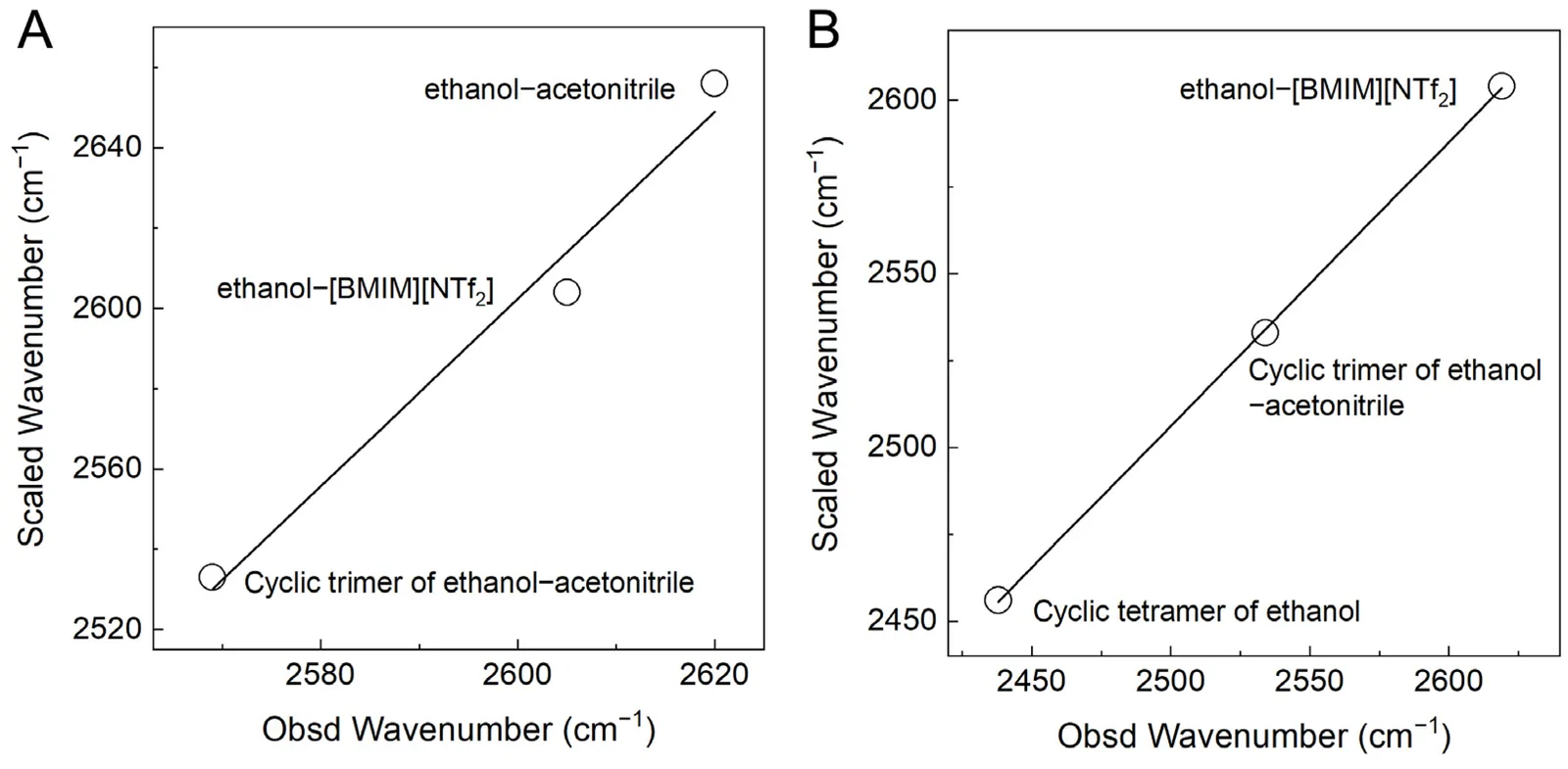
The correlation between the scaled and observed wavenumbers of *ν*(O−D) at *x*(IL) = 0.02 (**A**) and *x*(IL) = 0.1 (**B**).

To further verify the spectral assignments, Table 1 summarizes the calculated and experimentally observed *ν*(O−D) stretching vibration frequencies. The *ν*(O−D) frequencies of the deuterated complexes were calculated with the Gaussian method and are listed in Table 1. The scaled wavenumbers were scaled by a factor of 0.947. For the cyclic ethanol tetramer, two scaled *ν*(O−D) frequencies with similar intensities are listed in Table 1, and their average was used in Figure 9. These calculated frequencies follow the order: ethanol−acetonitrile > ethanol−IL > cyclic ethanol trimer−acetonitrile > cyclic ethanol tetramer. This trend is in excellent agreement with the experimentally observed order.

It should be noted that the calculated frequency difference between the ethanol–IL and ethanol–acetonitrile complexes is significantly greater than the corresponding experimental difference. This discrepancy arises because the calculated frequencies correspond to isolated, fully optimized complexes in the gas phase, where the IL−ethanol interaction is strongly stabilized by multiple hydrogen bonds and electrostatic contacts. In the condensed phase, these interactions are partially screened by the surrounding solvent environment and by the formation of larger aggregates, which effectively reduces the spectral separation between the two types of complexes.

Both computational and experimental evidence collectively confirms that, in the ethanol-*d*_1_−acetonitrile, the positive peak at 2625 cm^−1^ is assigned to the ethanol-*d*_1_−acetonitrile interaction complex. In the ethanol-*d*_1_−acetonitrile−[BMIM][NTf_2_], this peak persists even at *x*(IL) = 0.02, indicating that the ethanol-*d*_1_−acetonitrile interaction has not been completely eliminated. At *x*(IL) = 0.1, the positive peak located near 2619 cm^−1^ is attributed to the ethanol-*d*_1_−IL interaction complex. Furthermore, Figure 9 shows a general trend between the scaled and observed values, which supports the reliability of the IR band assignments.

### 2.4. Molecular Intrinsic Cause of Azeotrope Elimination by [BMIM][NTf_2_]

Quantum chemical calculations were performed to investigate the interaction energies and hydrogen bond lengths of the complexes and to compare the strengths of the formed hydrogen bonds. The results showed that the ethanol−ethanol interaction is stronger than the ethanol−acetonitrile interaction. MD simulations were also carried out. Analysis of the RDF revealed that in the ethanol−acetonitrile system, the ethanol−ethanol interaction is stronger than the ethanol−acetonitrile interaction. Upon addition of the IL, the RDF peak for ethanol−acetonitrile decreased, indicating that the IL affects the ethanol−acetonitrile interaction. In addition, the RDF peak for ethanol−IL was higher than that for ethanol−acetonitrile, suggesting that the ethanol−IL interaction is stronger than the ethanol−acetonitrile interaction. In the infrared spectroscopy experiments, it was found that the addition of either acetonitrile or IL to ethanol induced a blue shift, indicating a weakening of hydrogen bond strength. However, the blue shift caused by IL was more pronounced, further indicating that the ethanol−IL interaction is stronger than the ethanol−acetonitrile interaction. This is consistent with the theoretical calculations. The experimental observations are in good agreement with the simulation results, both confirming that the ethanol−IL interaction is stronger than the ethanol−acetonitrile interaction. This enhanced interaction increases the relative volatility between the azeotropic components and, thus, enables separation of the azeotrope, which is also the intrinsic mechanism by which ionic liquids break the azeotropic system.

## 3. Experimental Section

### 3.1. Materials

Table 2 provides detailed information of ethanol-*d*_1_, acetonitrile, and [BMIM][NTf_2_]. To prevent spectral interference between the *ν*(O−H) band of ethanol and the *ν*(C2−H) band of [BMIM]^+^, ethanol-*d*_1_ was employed in place of ethanol. The [BMIM][NTf_2_] was stored in a desiccator to avoid water absorption.

### 3.2. Sample Preparation

Binary mixtures of ethanol-*d*_1_−acetonitrile and ternary mixtures of ethanol-*d*_1_–acetonitrile–[BMIM][NTf_2_] with various compositions were prepared by weighing. For the binary mixtures, the mole fraction of ethanol-*d*_1_ was decreased from 0.9 to 0.1 with an interval of 0.1. For the ternary mixtures, the mole fraction of [BMIM][NTf_2_] was fixed at 0.02 and 0.1, while the mole fraction of ethanol-*d*_1_ was varied from 0.8 to 0.1 with a decrement of 0.1.

### 3.3. Fourier-Transform Infrared Spectroscopy (FTIR) and Excess Spectroscopy

A Nicolet (Madison, WI, USA) iS50 FTIR spectrometer with a Mercury Cadmium Telluride detector was employed for all experiments. The measurements were performed at ambient temperature (~25 °C) using an Attenuated Total Refraction (ATR) accessory with a ZnSe crystal, which enables 12 internal reflections at an incident angle of 45°. The ATR correction was performed according to the formula proposed by Hansen [29]. The refractive index was measured with an Abbe refractometer. To improve the spectral resolution of infrared spectroscopy, excess spectroscopy was adopted. The excess spectrum is defined as the difference between the experimental spectrum of the mixture and its ideal spectrum [30,31]. The calculation formula can be expressed in terms of excess molar absorbance (*ε*^E^) as follows:

(1) $$\varepsilon^{E}=\frac{A}{d\left(\sum_{i=1}^{n}Ci\right)}-\left(\sum_{i=1}^{n}x_{i}\varepsilon_{i}^{*}\right)$$

where *ε*^E^ is the excess molar absorbance, *A* is the absorbance of the mixture, *d* is the light path length, *C_i_*, *x_i_*, and *ε_i_* are the molar concentration, mole fraction, and molar absorption coefficient of the component *i* in its pure component state, respectively.

Subtracting the ideal spectrum from the actual spectrum yields positive and negative peaks, which represent species with increased and decreased relative amounts, respectively.

### 3.4. Quantum Chemical Calculations

Gaussian 16 software [32,33] and M06−2X/6−311++G** method [34] were used for quantum chemical calculations. The default integration grid in Gaussian 16 was employed throughout. The SCF convergence criterion was set to 10^−8^ Hartree (SCF = Tight) to ensure well-converged wavefunctions. All optimized structures were verified as true local minima by the absence of imaginary frequencies. The procedure consisted of three steps: (1) The initial monomers were drawn using ChemDraw (version 23.1.1). Each monomer was individually optimized to identify its most stable conformation. (2) The most stable monomer structure was placed at all possible interaction sites of another monomer to form complex configurations. The resulting complex configurations were then optimized to obtain the most stable configuration. This procedure was repeated iteratively to obtain more multimers. (3) Basis set superposition error corrections were subsequently applied to the optimized complexes, and the frequency was corrected using the frequency correction factor (0.947), using the standard Counterpoise method developed by Boys and Bernardi [35,36]. The structure with the maximum absolute interaction energy was chosen as the most stable configuration. Zero-point energy (ZPE) corrections, obtained from the harmonic frequency calculations, were included in all reported binding and interaction energies. Gibbs free energies were calculated, and interaction energies included thermal corrections.

### 3.5. MD Simulations

MD simulations were conducted using GROMACS 2022.3 [37] at 298.15 K and 1 bar. In the pseudo-binary systems, the mole fractions of the components were the same as those in the corresponding binary system, defined as follows:

$$\begin{array}{l} x_{1}^{\prime }=\frac{n_{1}}{n_{1}+n_{2}}\\ x_{2}^{\prime }=\frac{n_{2}}{n_{1}+n_{2}}\\ x_{3}=\frac{n_{3}}{n_{1}+n_{2}} \end{array}$$

where *n*_1_, *n*_2_, and *n*_3_ represent the number of molecules of ethanol, acetonitrile, and [BMIM][NTf_2_], respectively. The binary system contained 1000 molecules in total. For the pseudo-binary system, 100 ion pairs of [BMIM][NTf_2_] were added to maintain a mole fraction of *x*_3_ = 0.1, corresponding to the minimum experimental concentration of the IL. Initial configurations for mixtures at various concentrations were generated with the Packmol package [38], corresponding to acetonitrile mole fractions of 0.10, 0.30, 0.50, and 0.70. Topology files were built from the General Amber Force Field [39] using Sobtop [40], and atomic charges were derived via the RESP method with the Multiwfn software (version 3.8 (dev)) [41,42]. The force field was tested by comparing simulated densities at 298.15 K with experimental data. As listed in Table S3, the simulated and literature values differ by 2.73%. This small difference confirms that the force field is dependable and produces accurate results. Long-range electrostatic contributions were accounted for via the particle mesh Ewald [43,44]. A cutoff distance of 1.0 nm was set for nonbonded interactions. The simulation process consists of the following steps: (1) energy minimization through the steepest descent algorithm; (2) a 10 ns equilibration phase in the NPT ensemble; (3) after the temperature, density, and total energy had converged to stable values, a 20 ns NPT trajectory was generated and used for subsequent data analysis. Equilibrium was verified by tracking how temperature, density, and total energy evolved with time, as shown in Figures S4 and S5. SDF was subsequently obtained with the VMD (version 1.9.3) [45] and TRAVIS (version 190101) [46] software.

## 4. Conclusions

This study employs a combined approach of infrared spectroscopy experiments and theoretical calculations to elucidate the molecular-level mechanism by which [BMIM][NTf_2_] breaks the ethanol−acetonitrile azeotropic system. The investigation focuses primarily on the microstructures, interactions, and dynamic behavior of the system.

Spectral analysis identified the spectroscopic characteristics of the system before and after azeotrope elimination and assigned the observed peaks to possible interaction complexes. An acetonitrile−ethanol interaction complex was identified in both the acetonitrile−ethanol and the acetonitrile−ethanol−[BMIM][NTf_2_] (*x*(IL) = 0.02) systems. At *x*(IL) = 0.1, it cannot be resolved. Comparing the spectra shows that adding the IL can disrupt this complex. Quantum chemical calculations and MD simulations consistently showed that the ethanol−IL interaction is stronger than the ethanol−acetonitrile interaction.

In summary, the microscopic mechanism by which the IL breaks the azeotrope is as follows: the ionic liquid forms stronger interaction complexes with ethanol, disrupting the original ethanol−acetonitrile complexes, increasing the relative volatility of the azeotropic components, and ultimately achieving their separation. This work provides a basis for understanding azeotrope breaking by ILs and offers insights for the separation of azeotropic mixtures.

## Figures and Tables

**Table 1:** Calculated and scaled *ν*(O−D) frequencies (cm^−1^) of possible complexes, together with experimental observed *ν*(O−D) frequencies. The frequencies were scaled by 0.947.

Complex	Calculated *ν*(O−D)	Scaled *ν*(O−D)	Observed *ν*(O−D)
ethanol−acetonitrile	2805	2656	2620
[BMIM][NTf_2_]− ethanol	2750	2604	2605 2619
Cyclic trimer of ethanol−acetonitrile	2675	2533	2569 2534
Cyclic tetramer of ethanol	2578 2610	2441 2471	2432 2438

**Table 2:** Information on the materials used.

Sample	Purity (%)	Company
Ethanol-*d*_1_	99.5%	Shanghai Macklin Biochemical Co., Ltd. (Shanghai, China)
Acetonitrile	99.8%	Sinopharm Chemical Reagent Co., Ltd. (Shanghai, China)
[BMIM][NTf_2_]	99.0%	Shenyang Cynosure Technologies Co. Ltd. (Shenyang, China)

## Data Availability

The original contributions presented in the study are included in the article and Supplementary Materials, further inquiries can be directed to the corresponding author.

## Supplementary Materials

The following supporting information can be downloaded at: https://www.mdpi.com/article/10.3390/molecules31183345/s1. Reference [47] is cited in the Supplementary Materials.

## References

[1] Zhong P., Zhang L., Luo N., Liu J. (2023). Advances in the Application of Acetonitrile in Organic Synthesis since 2018. Catalysts.

[2] Maya J., Malloum A., Fifen J.J., Dhaouadi Z., Ekobena Fouda H.P., Conradie J. (2025). Acetonitrile Liquid Phase Modeling by the Quantum Cluster Equilibrium Theory. Isr. J. Chem..

[3] Lei Z., Dai C., Zhu J., Chen B. (2014). Extractive Distillation with Ionic Liquids: A Review. AIChE J..

[4] Meirelles A., Weiss S., Herfurth H. (1992). Ethanol Dehydration by Extractive Distillation. J. Chem. Technol. Biotechnol..

[5] Ma S., Shang X., Zhu M., Li J., Sun L. (2019). Design, Optimization and Control of Extractive Distillation for the Separation of Isopropanol-Water Using Ionic Liquids. Sep. Purif. Technol..

[6] Shen T., Teng L., Hu Y., Shen W. (2023). Systematic Screening Procedure and Innovative Energy-Saving Design for Ionic Liquid-Based Extractive Distillation Process. Front. Chem. Sci. Eng..

[7] Chaban V.V. (2025). Solubility Properties of Methanol in Organic Solvents. Comprehensive Methanol Science.

[8] Pereiro A.B., Araújo J.M.M., Esperança J.M.S.S., Marrucho I.M., Rebelo L.P.N. (2012). Ionic Liquids in Separations of Azeotropic Systems—A Review. J. Chem. Thermodyn..

[9] Cheng Y., Wang J., Gao D., Zhang H., Kwon Y.M., Li C., Li J., Wang X., Liu A., Zhang L. (2025). Industrial Applications of Ionic Liquids in Acetonitrile–Water Azeotrope Separation: Molecular Dynamics and DFT Insights into Azeotrope Breaking Methods. Ind. Eng. Chem. Res..

[10] Zhu Z., Geng X., He W., Chen C., Wang Y., Gao J. (2018). Computer-Aided Screening of Ionic Liquids As Entrainers for Separating Methyl Acetate and Methanol via Extractive Distillation. Ind. Eng. Chem. Res..

[11] Janakey Devi V.K.P., Sai P.S.T., Balakrishnan A.R. (2018). Ammonium-Based Ionic Liquid as an Entrainer for the Separation of *n* -Propanol + Water and Isopropanol + Water Mixtures. J. Chem. Eng. Data.

[12] Gjineci N., Boli E., Tzani A., Detsi A., Voutsas E. (2016). Separation of the Ethanol/Water Azeotropic Mixture Using Ionic Liquids and Deep Eutectic Solvents. Fluid Phase Equilibria.

[13] Boli E., Dimou E., Voutsas E. (2018). Separation of the Isopropanol-Water Azeotropic Mixture Using Ionic Liquids. Fluid Phase Equilibria.

[14] Xing Y., Cui X., Xu S., Feng T., Zhang X., He J., Wang J. (2021). Isobaric Vapor–Liquid Equilibrium for Methyl Acetate + Methanol with Double Salt Ionic Liquid [EMIM][Cl]0.5[DCA]0.5 as Entrainer at 101.3 kPa. Fluid Phase Equilibria.

[15] Zheng Y.-Z., Jiang Y.-X., Zhou Y., Zhang Y.-C. (2023). The Molecular Nature of the Eliminating Azeotropy of Dimethyl Carbonate–Ethanol System by Ionic Liquid Entrainer. Sep. Purif. Technol..

[16] Winnert J.M., Devi V.K.P.J., Brennecke J.F. (2019). Using Dialkylimidazolium Ionic Liquids To Break the Methanol + Methyl Acetate Azeotrope. Ind. Eng. Chem. Res..

[17] Chai Y., Zheng X.-P., Du Y.-P., Zhou Y., Zheng Y.-Z. (2024). A Combination of FTIR and DFT to Study the Microstructure Properties of Ionic Liquid–Acetonitrile–Methanol Systems. Spectrochim. Acta. A Mol. Biomol. Spectrosc..

[18] Yin Y., Liu S., Li X., Zhao C., Zhang W., Sun C., Yu Z., Xu X. (2026). From Experimental Exploration to Mechanistic Insight: Separation Mechanism of Ionic Liquids for Methanol-Acetonitrile Azeotrope. Chem. Phys..

[19] Fan H., Zhou H., Wu Z., Chen Y., Li Q., Zhao H. (2026). Isobaric Vapor–Liquid Equilibrium Experiments and Thermodynamic Correlations of Methyl Acetate + Methanol Containing Different Ionic Liquids at 101.3 kPa. J. Chem. Eng. Data.

[20] Floisand D.J., Miller T.C., Corcelli S.A. (2019). Dynamics and Vibrational Spectroscopy of Alcohols in Ionic Liquids: Methanol and Ethanol. J. Phys. Chem. B.

[21] Chen F., Zhang L., Liu Z., Yu G. (2020). Cluster Formation and Its Role in the Elimination of Azeotrope of the Acetone–Methanol Mixture by Ionic Liquids. Ind. Eng. Chem. Res..

[22] Liu S., Du X., Ji K., Lv L., Zhou Y. (2025). Mechanism of Azeotrope Elimination in the Ethyl Propionate–Ethanol System Using Ionic Liquid HMIMOAc as an Entrainer: Experimental and Theoretical Insights. Phys. Chem. Chem. Phys..

[23] Zhang Z., Zhang D., Li W., Zhang T., Wu K., Yang R., Zhang Q. (2018). Separation of Acetonitrile + Ethanol Mixture Using Imidazolium—Based ionic liquids as Entrainers. Fluid Phase Equilibria.

[24] Yue K., Lu J., Chen P., Gu S., Zhou G. (2024). Vapor–Liquid Equilibrium for Acetonitrile + Ethanol with Imidazole-Based Ionic Liquids as Entrainers at 101.3 kPa. J. Chem. Eng. Data.

[25] Hermansson K. (2002). Blue-Shifting Hydrogen Bonds. J. Phys. Chem. A.

[26] Hobza P., Havlas Z. (2000). Blue-Shifting Hydrogen Bonds. Chem. Rev..

[27] Arunan E., Desiraju G.R., Klein R.A., Sadlej J., Scheiner S., Alkorta I., Clary D.C., Crabtree R.H., Dannenberg J.J., Hobza P. (2011). Defining the Hydrogen Bond: An Account (IUPAC Technical Report). Pure Appl. Chem..

[28] Bawn C.E.H. (1940). The Hydrogen Bond. Nature.

[29] Hansen W.N. (1965). Expanded Formulas for Attenuated Total Reflection and the Derivation of Absorption Rules for Single and Multiple ATR Spectrometer Cells. Spectrochim. Acta.

[30] Li Q., Wang N., Zhou Q., Sun S., Yu Z. (2008). Excess Infrared Absorption Spectroscopy and Its Applications in the Studies of Hydrogen Bonds in Alcohol-Containing Binary Mixtures. Appl. Spectrosc..

[31] Zhang Y., Wu Z., Wang Y., He H., Yu Z. (2020). Excess Spectroscopy and Its Applications in the Study of Solution Chemistry. Pure Appl. Chem..

[32] Frisch M.J., Pople J.A., Binkley J.S. (1984). Self-Consistent Molecular Orbital Methods 25. Supplementary Functions for Gaussian Basis Sets. J. Chem. Phys..

[33] Frisch M.J., Trucks G.W., Schlegel H.B., Scuseria G.E., Robb M.A., Cheeseman J.R., Scalmani G., Barone V., Petersson G.A., Nakatsuji H. (2016). Gaussian 16 Rev. C.01.

[34] Zhao Y., Truhlar D.G. (2008). The M06 Suite of Density Functionals for Main Group Thermochemistry, Thermochemical Kinetics, Noncovalent Interactions, Excited States, and Transition Elements: Two New Functionals and Systematic Testing of Four M06-Class Functionals and 12 Other Functionals. Theor. Chem. Acc..

[35] Kashinski D.O., Chase G.M., Nelson R.G., Di Nallo O.E., Scales A.N., VanderLey D.L., Byrd E.F.C. (2017). Harmonic Vibrational Frequencies: Approximate Global Scaling Factors for TPSS, M06, and M11 Functional Families Using Several Common Basis Sets. J. Phys. Chem. A.

[36] Boys S.F., Bernardi F. (1970). The Calculation of Small Molecular Interactions by the Differences of Separate Total Energies. Some Procedures with Reduced Errors. Mol. Phys..

[37] Bauer P., Hess B., Lindahl E. GROMACS 2022.3 Manual, 2022. https://zenodo.org/records/7037337.

[38] Martínez L., Andrade R., Birgin E.G., Martínez J.M. (2009). P ACKMOL: A Package for Building Initial Configurations for Molecular Dynamics Simulations. J. Comput. Chem..

[39] Wang J., Wolf R.M., Caldwell J.W., Kollman P.A., Case D.A. (2004). Development and Testing of a General Amber Force Field. J. Comput. Chem..

[40] Lu T. Sobtop, Version 1.0(Dev5). http://sobereva.com/soft/Sobtop.

[41] Lu T. (2024). A Comprehensive Electron Wavefunction Analysis Toolbox for Chemists, Multiwfn. J. Chem. Phys..

[42] Lu T., Chen F. (2012). Multiwfn: A Multifunctional Wavefunction Analyzer. J. Comput. Chem..

[43] Darden T., York D., Pedersen L. (1993). Particle Mesh Ewald: An *N*⋅log(*N*) Method for Ewald Sums in Large Systems. J. Chem. Phys..

[44] Essmann U., Perera L., Berkowitz M.L., Darden T., Lee H., Pedersen L.G. (1995). A Smooth Particle Mesh Ewald Method. J. Chem. Phys..

[45] Humphrey W., Dalke A., Schulten K. (1996). VMD: Visual Molecular Dynamics. J. Mol. Graph..

[46] Brehm M., Thomas M., Gehrke S., Kirchner B. (2020). TRAVIS—A Free Analyzer for Trajectories from Molecular Simulation. J. Chem. Phys..

[47] Salgado J., Regueira T., Lugo L., Vijande J., Fernández J., García J. (2014). Density and Viscosity of Three (2,2,2-Trifluoroethanol + 1-Butyl-3-Methylimidazolium) Ionic Liquid Binary Systems. J. Chem. Thermodyn..

