# Supplementary material for: Molecular Mechanism of Ethanol−Acetonitrile Azeotrope Disruption by the Ionic Liquid [BMIM][NTf2]
Source: Molecules. 2026 Sep 21;31(18):3345. doi: 10.3390/molecules31183345 (PMC13610555; doi:10.3390/molecules31183345)
Supplement: Supplementary file 1 [file molecules-31-03345-s001.zip › molecules-4467668-supplementary.pdf]

Supplementary Information

Molecular Mechanism of Ethanol−Acetonitrile Azeotrope Disruption by the Ionic Liquid [BMIM][NTf_2]

Pingxiao Jia[a], Sainan Wen[a], Xiuyu Du[a], Kuan Ji[a], Jiao Ma[a], Yilin Lyu[a], Yu Zhou*[,a]

[a] *Shandong Sino-Japanese Center for Collaborative Research of Carbon Nanomaterials, College of Chemistry and Chemical Engineering, Qingdao University, Qingdao 266071, China.*

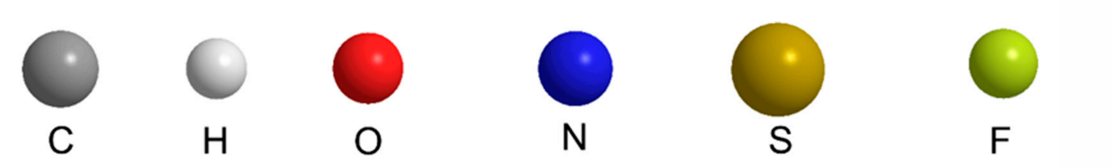

Fig. S1 The colors of the atoms in the configurations represent different chemical elements.

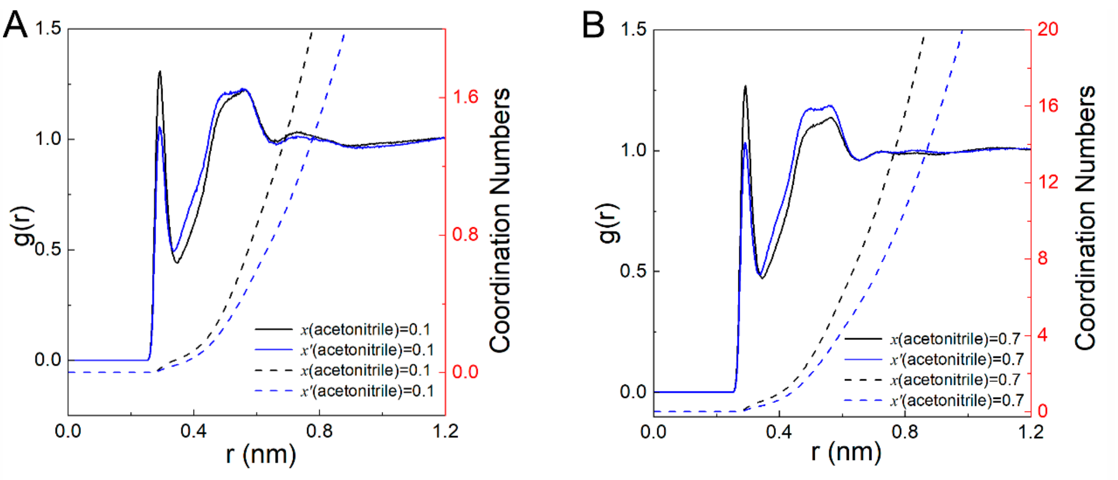

Fig. S2 RDF and coordination number of ethanol−acetonitrile without and with IL at *x*(acetonitrile) = 0.1(A), 0.7(B). The *x* denotes the absence of IL, and *x*' denotes the RDF obtained at *x*(IL) = 0.1. Solid line: RDF g(r); dash line: Coordination numbers.

Table S1 Coordination numbers of acetonitrile around ethanol.

x(acetonitrile)	Without IL Coordination numbers	With IL Coordination numbers
0.1	0.078	0.037
0.7	0.573	0.269

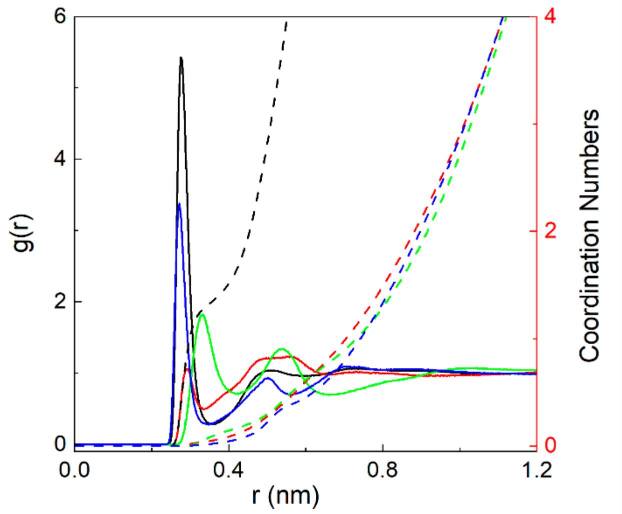

Fig. S3 RDF and coordination numbers of ethanol, acetonitrile, $[BMIM]^+$, $[NTf2]^-$ around the ethanol with IL at *x*(acetonitrile) = 0.1. Solid line: RDF g(r); dash line: Coordination numbers. Black: ethanol−ethanol; Red: ethanol−acetonitrile; Blue:

ethanol−[NTf_2]$^-$; Green: ethanol−[BMIM]$^+$.

Table S2 Coordination numbers (CN) of ethanol, acetonitrile, [BMIM]$^+$, [NTf2]$^-$ around the ethanol.

	ethanol−ethanol	ethanol−acetonitrile	ethanol−[NTf_2]$^-$	ethanol−[BMIM]$^+$
CN	1.35	0.037	0.166	0.015

Table S3 Comparison of simulated and experimental densities (g/cm^3) of pure [BMIM][NTf_2] at 298.15 K.

Pure IL	Temperature	Simulation	Experiment	Errors
[BMIM][NTf_2]	298.15 K	1.4760 g/cm^3	1.4368 g/cm^3[1]	2.73%

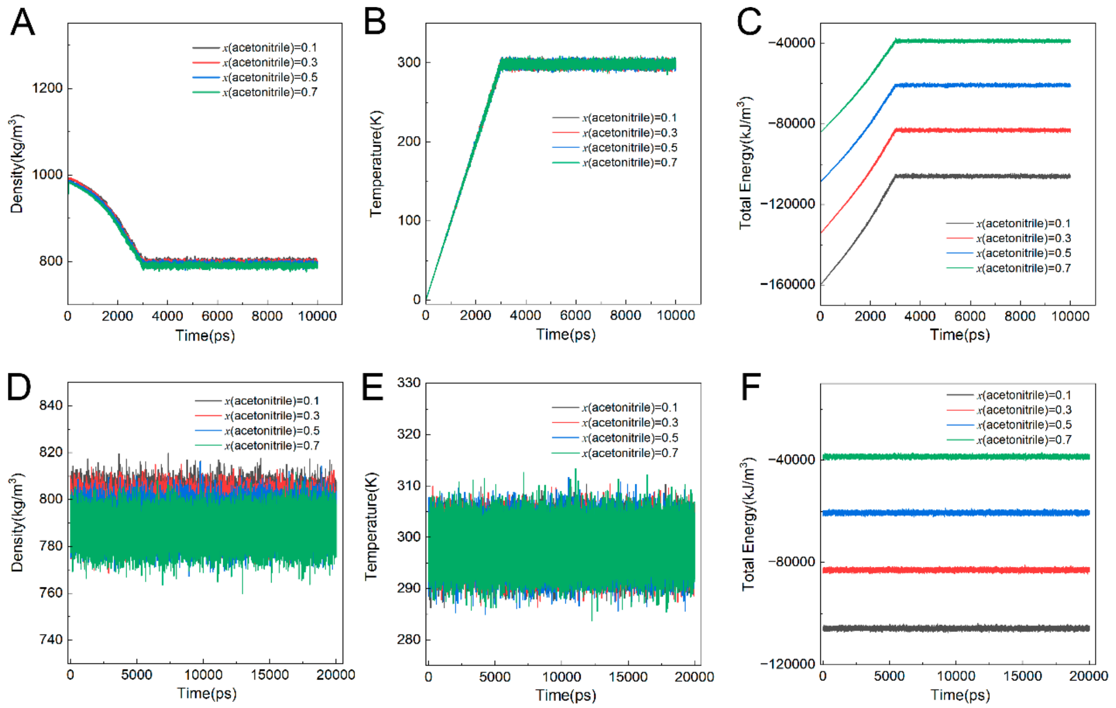

Fig. S4 Density (A), Temperature (B) and Total Energy (C) in the ethanol−acetonitrile mixtures calculated by 10 ns NPT equilibration trajectories. Density (D), Temperature (E) and Total Energy (F) in the ethanol−acetonitrile mixtures calculated by an additional 20 ns NPT−MD simulation for data analysis. x(acetonitrile) = 0.1, 0.3, 0.5 and 0.7.

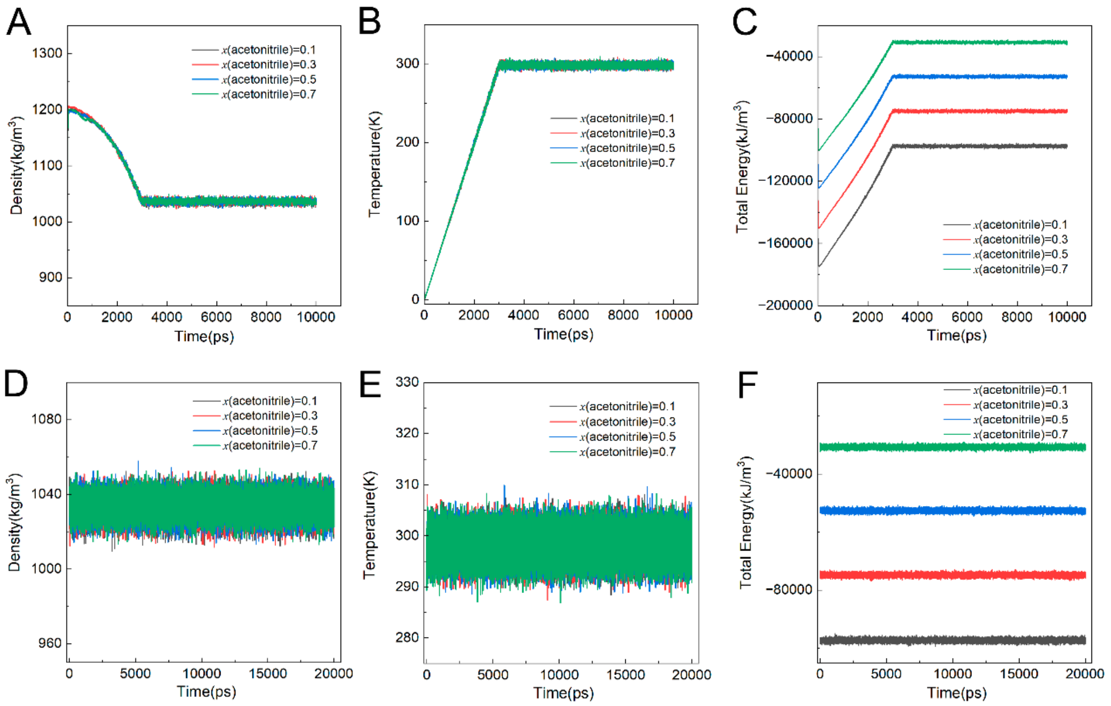

Fig. S5 Density (A), Temperature (B) and Total Energy (C) in the ethanol−acetonitrile−[BMIM][NTf2] mixtures calculated by 10 ns NPT equilibration trajectories. Density (D), Temperature (E) and Total Energy (F) in the ethanol−acetonitrile−[BMIM][NTf2] mixtures calculated by an additional 20 ns NPT−MD simulation for data analysis. *x*(acetonitrile) = 0.1, 0.3, 0.5 and 0.7.

1. Salgado, J.; Regueira, T.; Lugo, L.; Vijande, J.; Fernández, J.; García, J. Density and Viscosity of Three (2,2,2-Trifluoroethanol + 1-Butyl-3-Methylimidazolium) Ionic Liquid Binary Systems. *The Journal of Chemical Thermodynamics* **2014**, *70*, 101–110, doi:10.1016/j.jct.2013.10.027.
